# Effectiveness and cost-effectiveness of an exposure-based return-to-work programme for patients on sick leave due to common mental disorders: design of a cluster-randomized controlled trial

**DOI:** 10.1186/1471-2458-9-140

**Published:** 2009-05-13

**Authors:** Erik Noordik, Frank J van Dijk, Karen Nieuwenhuijsen, Jac JL van der Klink

**Affiliations:** 1 Coronel Institute of Occupational Health, Academic Medical Center (AMC), University of Amsterdam, PO Box 22700, 1100 DE, Amsterdam, The Netherlands; 2 University Medical Center Groningen, Antonius Deusinglaan 1, 9713 AV, Groningen, The Netherlands

## Abstract

**Background:**

To reduce the duration of sick leave and loss of productivity due to common mental disorders (CMDs), we developed a return-to-work programme to be provided by occupational physicians (OPs) based on the principles of exposure in vivo (RTW-E programme). This study evaluates this programme's effectiveness and cost-effectiveness by comparing it with care as usual (CAU). The three research questions we have are: 1) Is an RTW-E programme more effective in reducing the sick leave of employees with common mental disorders, compared with care as usual? 2) Is an RTW-E programme more effective in reducing sick leave for employees with anxiety disorders compared with employees with other common mental disorders? 3) From a societal perspective, is an RTW-E programme cost-effective compared with care as usual?

**Methods/design:**

This study was designed as a pragmatic cluster-randomized controlled trial with a one-year follow-up and randomization on the level of OPs. We aimed for 60 OPs in order to include 200 patients. Patients in the intervention group received the RTW-E programme. Patients in the control group received care as usual. Eligible patients had been on sick leave due to common mental disorders for at least two weeks and no longer than eight weeks. As primary outcome measures, we calculated the time until full return to work and the duration of sick leave. Secondary outcome measures were time until partial return to work, prevalence rate of sick leave at 3, 6, 9, and 12 months' follow-up, and scores of symptoms of distress, anxiety, depression, somatization, and fatigue; work capacity; perceived working conditions; self-efficacy for return to work; coping behaviour; avoidance behaviour; patient satisfaction; and work adaptations. As process measures, we used indices of compliance with the intervention in the intervention group and employee-supervisor communication in both groups. Economic costs were calculated from a societal perspective. The total costs consisted of the costs of consuming health care, costs of production loss due to sick leave and reduced productivity, and out-of-pocket costs of patients for travelling to their OP.

**Discussion:**

The results will be published in 2009. The strengths and weaknesses of the study protocol are discussed.

**Trial registration:**

ISRCTN72643128

## Background

### Common mental disorders and the effectiveness of interventions

Common mental disorders (CMDs) are described as mild to moderately severe mental disorders. However, there is no generally accepted definition. Some experts define CMDs as consisting of depressive, anxiety, and somatoform disorders based on DSM IV criteria [1]. Others define CMDs as having a depressive episode or one of four different anxiety disorders defined by ICD-10, including mixed anxiety and depressive disorder with sub-threshold symptoms [2], or define CMDs as ranging from stress symptoms, measured by for instance the General Health Questionnaire, to minor usually mixed anxiety and depressive symptoms as often seen in primary care [3]. We defined CMDs as a broad concept including stress-related disorders and depressive, anxiety, and adjustment disorders based on DSM IV criteria.

The working population prevalence rates of anxiety, depressive disorders, and stress-related disorders are high. Sanderson et al. [4] showed in a review that the working population prevalence rates vary by region. They found prevalence rates of depressive disorders varying from 2.2% in Australia to 4.8% in the Netherlands. Also, the prevalence rates of various anxiety disorders vary, such as from 0.1% in Australia to 1.8% in the US for agoraphobia, and from 5.2% in the US to 5.6% in the Netherlands for simple phobia. The prevalence rates of stress-related disorders, measured by the General Health Questionnaire (GHQ) or Maslach Burnout Inventory (MBI-GS) vary from 35% in the UK [3] to 27% in Finland [5].

The impact of CMDs on personal lives, companies, and society is substantial. CMDs are associated with personal costs such as a reduction in the quality of life, role functioning, and income. Furthermore, CMDs are associated with increased sick leave [2-5] and loss of productivity [4,6]. At a societal level, the estimated costs due to CMDs are deemed to be substantial. The annual costs of depressive disorders in the United States were estimated at $83.1 billion in 2000 [7]. A major proportion (62%) of these costs is due to loss of productivity, sickness absence, and work disability. Although there is a lack of cost-of-illness data related to anxiety disorders in Europe [8], some estimates are available from individual countries. The annual excess costs of prevalent cases of mood and anxiety disorders at population level in the Netherlands are estimated at 560 million euros [9] per 1 million people aged 18 to 65 years. Of these costs, 85% are due to production loss.

Evaluation studies of clinical treatments for various anxiety and depressive disorders, such as cognitive behavioural therapy (CBT) and pharmacotherapy, showed that symptoms can be reduced effectively [10-13]. However, only a limited number of studies measured work-related outcomes [14] and hardly any studies demonstrated effectiveness in terms of reduced sick leave or increased productivity. We may therefore conclude that well-known evidence-based treatments for depressive and anxiety disorders such as CBT and medication can be effective in reducing symptoms but do not automatically reduce absenteeism or improve productivity. In order to achieve earlier return to work and build up productivity, work-directed interventions seem promising, especially when work conditions can be considered as causal factors.

Van der Klink et al. [15] and Schene et al. [16] both conducted a randomized controlled trial (RCT) to evaluate the effectiveness of a work-directed intervention programme with workers on sick leave due to stress-related and major depressive disorders, respectively. They compared the intervention programme with care as usual. In both studies, they found a reduced duration of sick leave of a work-directed intervention programme. The mean duration of sick leave was reduced by 21 [15] and 92 days [16], respectively. Moreover, Blonk et al. [17] found, in a RCT on the effectiveness of an similar work-directed intervention programme for self-employed workers, a reduced median duration of sick leave of approximately 200 days, compared with a no treatment and a regular CBT control group. Furthermore, studies on the effectiveness of similar but not work-directed intervention programmes [18,19] showed no reduction of the duration of sick leave. So, there are indications that work-directed interventions can reduce sick leave more effectively.

The above-described work-directed interventions [15-17] combined an activating problem-solving approach to perceived stressors and restoring contact with the working environment. These ingredients appear to contribute to a shorter duration of sick leave. For the activating problem-solving approach, this presumption is supported by the findings of Van Rhenen et al. [20]. They found, in a large cohort study of employees with a high stress level, that employees with an active problem-solving coping strategy are less likely to drop out because of sickness absence in terms of frequency, length, and duration of sickness absence. Moreover, an avoidant coping style was associated with increased frequency of reporting sickness and the duration of sick leave.

Restoring contact with the working environment by a gradual increase in working hours could be another effective ingredient of work-directed intervention programmes that contributes to a shorter duration of sick leave, as the studies described above [15-17] also have this element in common. In order to increase working hours gradually during the return to work process and prevent avoidance of perceived stressors simultaneously, exposure in vivo as part of a work-directed intervention programme may be an interesting new approach. During such an exposure in vivo treatment patients can gradually learn to cope more actively with stressful work situations.

Exposure in vivo is a well-documented, evidence-based behavioural treatment for anxiety disorders [21], which decreases anxiety symptoms and associated avoidance behaviour [22,23]. Although high quality studies on work-related effects of exposure in vivo in patients with anxiety disorders are scarce [24], the available evidence suggests that exposure in vivo can have neutral or positive work-related effects on patients with obsessive-compulsive disorder (OCD) and posttraumatic stress disorder (PTSD) [24-30]. Exposure in vivo treatment in only one of these controlled studies [30] can be considered as being work-directed. The effectiveness of this intervention indicated a high re-employment rate of PTSD patients, a result that was also found in another non-controlled study with PTSD patients [31]. So, exposure in vivo could be an effective part of a work-directed intervention programme. However, none of these studies reported the effects on sick leave.

In order to enhance the return to work of patients on sick leave due to CMDs, we developed a work-directed intervention programme based on the principles of exposure in vivo (RTW-E programme). We developed this programme for application by OPs in addition to their care as usual for patients with CMDs. To evaluate the effectiveness of the programme on sick leave measures, we planned to compare this programme with care as usual. As part of this evaluation, we also planned to compare the effectiveness of the RTW-E programme on sick leave measures between employees with anxiety disorders and employees with other CMDs. We consider this evaluation interesting as exposure in vivo in clinical treatment is an established treatment in reducing anxiety symptoms. In addition, we planned to evaluate the cost-effectiveness of the RTW-E programme compared with care as usual (CAU), from a societal perspective.

In the designed study, we therefore hypothesized:

- that an RTW-E programme is more effective in reducing the sick leave of employees with CMDs compared with CAU

- that an RTW-E programme is more effective in reducing sick leave for employees with anxiety disorders compared with employees with other CMDs

- that, from a societal perspective, an RTW-E programme is cost-effective compared with care as usual.

## Methods/design

To describe the design of the study, all the items of the CONSORT statement [32], aimed at improving the quality of reporting of RCTs, will be discussed. To describe the economic evaluation in this study, we used the guidelines on presenting studies on health economics [33].

### Dutch context

In the Netherlands, most employees on sick leave due to CMDs visit an occupational physician (OP). Individual employees and their employers have, according to national legislation, a shared responsibility for minimizing health complaints and sick leave. In order to accomplish this, the employer is obliged to contract an OP who is a medical expert as well as an advisor on sick leave for both the employee and the employer. For the individual employee and his supervisor, an important task of the OP is individual counselling in the case of sick leave in order to support the return to work. In order to structure the individual counselling of patients with CMDs, Dutch OPs have developed and implemented professional practice guidelines [34,35]. A majority of the Dutch OPs have received training in counselling according to these guidelines. Although compliance with these guidelines can be improved [36], we considered working in accordance with these guidelines as representative of care as usual (CAU) in the Netherlands.

### Study design

The study was designed as a two-armed cluster-randomized trial (RCT) with randomization on the level of OPs. OPs from various occupational health services agreed to participate in the study. The OPs in the control group counselled their patients according to CAU and the OPs in the intervention group counselled their patients according to the RTW-E programme. After inclusion and baseline measurement, measurement took place every three months from the first day of sick leave for a period of twelve months. In the intervention group, compliance with the RTW-E programme was monitored during follow-up. Sick leave was monitored continuously until full return to work was achieved. Recurrences of sick leave were measured every three months.

Patients were blinded to the intervention, as OPs were instructed not to inform patients about the content of their counselling. Blinding OPs to the intervention was not feasible as every OP was trained to apply either the experimental intervention (counselling according to the RTW-E programme) or CAU. Therefore, all the OPs knew which type of intervention they conducted. To prevent contamination between the two groups, the OPs in the control group were neither informed about nor received training in the RTW-E programme. The OPs in the intervention group were requested not to disclose the new intervention during the inclusion period to OPs in the control group. Furthermore, the chance of contamination was small as almost every OP in the study worked at a geographically different worksite.

**Figure 1:**
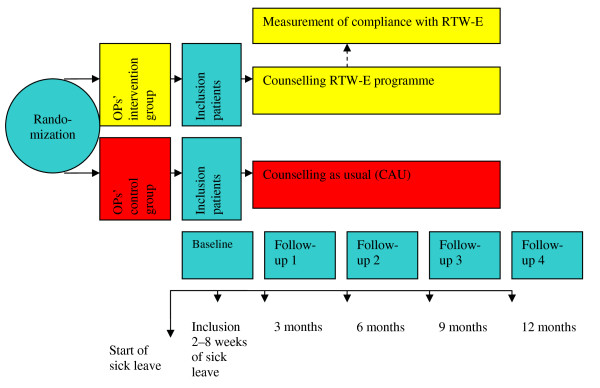
**Design of the study**.

The design we used is presented in Figure 1.

The Medical Ethics Committee of the Academic Medical Center and University of Amsterdam approved the study design, research protocol, information brochure, and informed consent. Patients voluntarily participated in the study. A written informed consent was obtained from each patient. Patients were informed about their right to withdraw from the study at any time, without specification of reasons and with no negative consequences for their treatment. Patients who withdrew received care as usual. The acronym we used for the study is 'WORK UP study'.

### Sample size and randomization

In this study, we wanted to include 60 OPs who would in turn include 200 patients in order to be able to detect a statistically significant difference in sick leave between groups. This sample size was not based on a power analysis as software for such an analysis of survival data was not available at the time. In a comparable intervention study of Van der Klink et al. [15], significant differences were found with 33 OPs (17 in the experimental and 16 in the control group) and 192 patients (109 experimental and 83 control group). In that study, each OP included an average of 6 patients (range 1–16).

After randomization, we expected the experimental and control groups each to consist of 30 OPs, who were expected to include 3 or 4 patients each. To realize the inclusion of 200 patients, we requested every OP to include 8 patients as we expected a high drop-out rate of OPs e.g. caused by a dynamic labour market.

OPs presented themselves for participation in the study. All the presented OPs were randomized to the experimental or control group. We performed a restricted randomization with blocks of four OPs. Every time four OPs presented themselves to researcher EN, these were randomized by researcher KN, concealed from researcher EN. After randomization, researcher KN informed EN about the allocation of every OP and saved the randomization file. If an OP in the control group dropped out within a month of randomization, we reused the attributed number of randomization. If an OP in the experimental group dropped out before being trained in the RTW-E programme, we reused the attributed number of randomization, as we wanted to prevent skewness in the distribution between the experimental and control groups as much as possible.

### Recruitment of occupational physicians

Participating OPs were recruited from May 2006 until August 2006 from various occupational health services geographically widespread over the Netherlands. The OPs were invited by a written letter accompanied by an information brochure in which the purpose, the demands, and the gains of the study were explained. After inclusion, the control group was offered a one-day training in the RTW-E programme if they had included the requested eight patients. For attending the training and each tutorial session, OPs received educational credits recognized by the National Association of Occupational Medicine. Before randomization, the OPs were committed to presenting eight patients for inclusion by signing an agreement. In addition, every OP received a seven-item questionnaire concerning personal (one item) and professional characteristics (seven items). We also committed the management of the occupational health services of participating OPs to the study by requesting them to sign an agreement in which they confirmed the feasibility of the study.

### Recruitment of patients

#### Inclusion criteria

We included patients absent from work due to CMDs if they were on sick leave for at least two weeks and no longer than eight weeks at the moment of inclusion. CMDs were defined as stress-related, adjustment, anxiety, or depressive disorders. Stress-related disorders were classified according to the guidelines for OPs [34,35]. Anxiety, depressive, and adjustment disorders were classified by the Diagnostic Statistical Manual (DSM), version IV. We included only patients who, at the moment of inclusion, had not yet fully returned to work.

#### Exclusion criteria

Patients with a current psychiatric disorder at the moment of inclusion, such as PTSS, substance-related disorder, or psychotic disorder as defined by DSM IV classification, were excluded. Also, patients with a primary somatic disorder according to the OP were excluded. Furthermore, non-Dutch speaking patients were excluded.

### Procedure

Patients who met the inclusion criteria according to the OP were invited to participate in the study. After a verbal invitation by the OP, patients received a written information leaflet about the study. The leaflet described the purpose and demands of the study, and contained an informed consent form. With the permission of the patient, the OP sent his name and telephone number to researcher EN, who contacted every patient by telephone to ask if they needed more information to decide whether or not to participate in the study.

If the patients decided to participate, they were requested to sign a written informed consent form, for participating in a telephone diagnostic interview of 25 minutes with researcher EN, to fill in a baseline questionnaire and 4 follow-up questionnaires at 3, 6, 9, and 12 months after reporting sick leave, respectively. Furthermore, patients gave informed consent to register daily, on weekly registration forms, their hours spent working, on sick leave, or on vacation, until they returned fully to work. After informed consent, researcher EN conducted a telephone diagnostic interview as soon as possible in order to check eligibility for inclusion in the study. Researcher EN informed the OPs about the inclusion or exclusion of a patient by sending them an email. If, during the diagnostic interview, patients were found to be suffering from a 'depressive episode', the risk of suicide was assessed using a protocol. In the case that we found a moderate or high suicidal risk, EN informed the patient and recommended the patient to inform their practitioner(s) about this. If the patient had not informed their practitioner(s) as we appointed, we informed the practitioner about the risk by ourselves if the severity of suicidal risk was deemed to be more important than the informed consent requirement.

After inclusion, the OPs in the intervention group counselled their patients according to the RTW-E programme and the OPs in the control group counselled their patients according to the CAU. After the OPs in the intervention group finished their counselling, they sent a copy of the filled-in homework assignment forms to researcher EN. These forms were used as indicators of compliance with the RTW-E programme.

### Intervention

#### Training OPs in the intervention group

The OPs in the intervention group received a two-day training in order to apply the RTW-E programme. Thereafter, three follow-up tutorial sessions were planned during the inclusion period. During these sessions, difficulties with applying the RTW-E programme were discussed with other participating OPs and practical solutions were exchanged for problems that had arisen. These sessions were guided by researchers EN or JvdK, or by an external well-informed psychologist.

#### Treatment of patients in the intervention group

After two or three weeks of sick leave, patients were informed about the RTW-E programme by their OP. Apart from an oral explanation about the rationale, aim, and procedure of the RTW-E programme, patients received a written patient information brochure about the programme. This information brochure can be downloaded in Dutch or English from . If the patients accepted the rationale and agreed with the aim and procedure of the RTW-E programme, the programme started. This means he or she learned to cope more actively by gradual exposure to work situations that evoke lower levels of anxiety, stress, or anger, instead of using avoidance behaviour in the case of a stressful work situation. This gradual exposure only concerns stressful work situations that can not be prevented or solved otherwise and are an intrinsic part of the job. For instance, a nurse who is anxious about injecting patients could start her exposure by watching a colleague who is injecting a patient, i.e. being exposed to a similar situation but with a lower level of perceived stress.

The patient is motivated and counselled by the OP in order to prepare, draw up, and evaluate an exposure-based RTW plan. This process is structured by giving patients several 'homework' assignments. The homework assignments and accompanying forms A to F support the patient to think about and describe:

a. the time he usually spends on different tasks in his job and the current feasibility of performing these tasks (form A),

b. a list of stressful work situations that are relevant to returning to work, the extent to which these work situations can be influenced, and how much he tends to avoid these work situations if they could not be prevented or solved otherwise (form B). The OP supports the patient in deciding which alternative (active) coping behaviour, instead of avoidance behaviour, will be more effective in reducing his negative feelings in the long term. Applying this alternative coping behaviour in the stressful work situation will serve as a goal in the RTW-E programme,

c. various work situations that are similar to the stressful work situation but with lower levels of perceived stress. After describing such similar work situations in as much detail as possible, the patient is invited to rank those work situations according to their perceived stressfulness. The result is called a stress hierarchy, in analogy to an anxiety hierarchy for anxiety-evoking situations used in exposure in vivo for anxiety disorders (form C),

d. realistic and acceptable RTW arrangements in cooperation with his supervisor. The RTW arrangements had to consist of a gradual increase in the amount of working hours, feasible tasks, and exposure to increasing levels of stress associated with the listed work situations in form C (form D). By practising the new coping behaviour in this way and experiencing several times that he is able to reduce his feelings of stress to an acceptable level without using avoidance behaviour, the patient will become more confident about his new coping behaviour. The gradual exposure is part of the return to work plan directly from the start of reintegration,

e. the evaluation of the RTW arrangements made in form D, in cooperation with his supervisor (form E),

f. new additional RTW arrangements in cooperation with his supervisor after evaluating the results of earlier RTW arrangements (form F).

The homework assignment forms A to F can also be downloaded in Dutch or English from .

#### Training OPs in the control group

We considered the Dutch guidelines on employees with CMDs [34,35] as care as usual. The OPs in the control group received a one-day training in order to update their skills in counselling according to these guidelines.

#### Treatment of patients in the control group

Patients in the control group received counselling according to care as usual (CAU). According to the guidelines, CMD is defined as a (temporary) lack of control of the patient in dealing with his demanding private, working, and health care environment. Therapy and guidance are aimed at helping the patient regain control and rebuild his social and occupational contacts and activities [35]. The OP can reach this goal by using recommended methods such as stress inoculation training, cognitive restructuring, graded activity, and time contingency during return to work.

CAU meant that patients were informed by their OP about the CAU, which could be supported by a patient information brochure, 'Nervous exhaustion'. This brochure can be downloaded from  (in Dutch). If the patient accepted the rationale of the CAU, the OP motivated the patient to prepare, draw up, and evaluate a RTW plan in co-operation with the supervisor. This RTW plan was based solely on a gradual and time-contingent increase in the amount of working hours and feasible tasks. It was not based on gradual exposure in vivo or on a stress hierarchy of work situations at the workplace. This RTW plan was also prepared and structured by giving patients several homework assignments aimed at strengthening the problem-solving behaviour of both the patient and his supervisor.

### Measures

#### Diagnostic interview

We used the Mini-International Neuropsychiatric Interview (MINI plus; Dutch version 5.0.0.) as a diagnostic interview in order to include or exclude patients [36]. To include patients, the MINI was administered for the classification of symptoms of major depression, dysthymia, panic disorder, agoraphobia, social phobia, simple phobia, obsessive-compulsive disorder, generalized anxiety disorder, hypochondria, and adjustment disorder according to Axis I of DSM IV. Other parts of the MINI were used to exclude patients. Psychometric properties of the MINI can be considered as good [37].

### Primary outcome

#### Sick leave

Time until full return to work and the duration of sick leave were used as primary outcome parameters. The time until full RTW was calculated as the number of calendar days from the first day of sick leave to the first day of full return to work. The duration of sick leave was calculated as the number of hours of calendar days on sick leave from the first day of sick leave until full return to work, lasting at least 28 calendar days without partial or full relapse.

### Secondary outcomes

#### Sick leave

As secondary measures of sick leave, we calculated the time until partial return to work and the prevalence rate of partial and full return to work at 3, 6, 9, and 12 months' follow-up. The time until partial RTW was calculated in a similar way to how we calculated time to full RTW. The prevalence rate was calculated as the number of persons who (partially or fully) returned to work divided by the total number of persons in the intervention or control group at 3, 6, 9, and 12 months' follow-up.

#### Distress, anxiety, depression, and somatization

We measured distress and somatization symptoms by using the distress and somatization subscales of the Four-Dimensional Symptom Questionnaire (4DSQ) [38]. Anxiety and depressive symptoms were measured by the anxiety and depression subscales of the 4DSQ and the Hospital Anxiety and Depression Scale (HADS).

The Four-Dimensional Symptom Questionnaire (4DSQ) is a Dutch self-report questionnaire that consists of 50 items distributed over 4 subscales. The distress subscale contains 16 items and the total score ranges from 0 to 32, the depression subscale contains 6 items and the total score ranges from 0 to 12, the anxiety subscale contains 12 items and the total score ranges from 0 to 24, and the somatization subscale contains 16 items and the total score ranges from 0 to 32. Higher scores indicate more distress, depression, anxiety, or somatization. The 4DSQ appears to be a valid and reliable self-report questionnaire for primary care patients. The Cronbach's alpha for the 4 subscales ranged from 0.84 to 0.90 [38].

The Hospital Anxiety and Depression Scale (HADS) is a 14-item self-report screening scale. It contains two 7-item subscales for anxiety and depression. Both subscale scores range from 0 to 21 with higher scores indicating more depression or anxiety. The HADS showed good homogeneity and reliability, with Cronbach's alpha for the subscales anxiety and depression ranged from 0.81 to 0.84 and from 0.79 to 0.86, respectively, in different Dutch samples [39].

#### Fatigue

We measured fatigue by using the multi-dimensional Checklist Individual Strength Questionnaire (CIS). CIS consists of 20 statements distributed over four dimensions: subjective feeling of fatigue (8 items), motivation (4 items), physical activity (3 items), and concentration (5 items). Each item is scored for the past 2 weeks on a 7-point Likert scale ranging from 1 (true) to 7 (not true). The CIS was able to measure changes in fatigue scores in groups as well as in individual workers in randomized controlled trials [40]. Furthermore, the discriminant validity of the CIS was adequate for employees in occupational groups [41]. The internal consistency of the CIS was also good for clinical and working populations, and the Cronbach's alpha for the total CIS was found to be 0.90 [42] and 0.91 [43], respectively.

#### Work capacity

We measured current work ability by using a single item of the Work Ability Index (WAI). We asked the patient to estimate their current work ability compared with their lifetime best. The score of this item ranges from 0 (cannot work at all) to 10 (best ever). The evidence for internal validity, predictive validity for disability, and test-retest reliability of the WAI questionnaire in workers was satisfactory [44,45].

#### Working conditions

We measured working conditions by using 9 out of the 14 subscales of the VBBA core questionnaire: a self-report questionnaire on perception and judgement of work. We used the subscales work pace and workload (11 items), emotional strain (7 items), decision latitude (8 items), autonomy (11 items), social support colleagues (11 items) and social support supervisor (11 items), ruminating (4 items), enjoyment of work (9 items), and job insecurity (4 items). The Cronbach's alpha for these scales ranged from 0.79 to 0.95, which we considered as good internal reliability [46]. The construct and concurrent validity of the VBBA core questionnaire are satisfactory [46].

#### Self-efficacy for return to work

We measured 'self-efficacy for return to work' by using a recently developed questionnaire. Jager [47] found, in a pilot study of workers on sick leave due to CMDs, a satisfactory construct validity and good reliability: the Cronbach's alpha was 0.94, and test-retest reliability 0.75.

#### Coping behaviour

We measured coping behaviour by using an adapted version of the shortened 19-item version of the original 30-item Utrecht Coping List (UCL) [48,49]. This self-report questionnaire was designed to measure the coping behaviour people use in stressful situations, life events, or daily hassles. We adapted the questionnaire so that all the items were concerned with problems at work. Each item was rated on a 4-point Likert scale ranging from 1 (never) to 4 (very often). The UCL includes 5 dimensions: (1) active problem-focusing (5 items), (2) seeking social support (5 items), (3) palliative reaction pattern (4 items), (4) avoidance behaviour (3 items), and (5) expression of emotions (2 items).

#### Avoidance behaviour related to work

To explore the concept of avoidance behaviour related to the return to work, we used two questions: Do you avoid emotional or stress-evoking work situations? Is your productivity negatively affected by this? Each question was scored on a 4-point Likert scale ranging from 1 (seldom or never) to 4 (very often).

#### Patient satisfaction with counselling of OP

We measured patient satisfaction with the counselling of the occupational physician by using the Patient Satisfaction with Occupational Health Services Questionnaire (PSOHQ) [50]. The questionnaire consists of 20 items and 4 subscales concerning 'being taken seriously as a patient', 'trust and confidentiality', 'expectations', and 'attitude towards occupational health services', respectively. The scales showed sufficient reliability [50].

#### Work adaptations

We measured work adaptations by using two questions: (1) have work adaptations been made during the last three months (yes/no)? and (2) if yes, specify these work adaptations.

### Process measures

#### Compliance of intervention

We measured the compliance of the counselling process of OPs and their patients with the RTW-E programme by scoring the presence and quality of filled-in homework assignment forms. The presence of filled-in forms A (Task Inventory) to F (Evaluation of Work Tasks and Practice Situations) was scored by yes or no. The quality of the filled-in forms was classified by two researchers (EN and KN) independently. The quality of each form was rated on a 3-point Likert scale: 0 = not in accordance with the purpose of the form, 1 = partly in accordance with the purpose of the form, and 2 = in accordance with the purpose of the form. The total quality score of compliance was classified for each patient as low (< 3), moderate (3–6), or high (> 6).

#### Communication employee-supervisor

We measured communication between the employee and supervisor by using a two-item questionnaire, derived from a standardized telephone interview concerning supervisor behaviour related to return to work [51].

#### Economic evaluation measures

In order to make a cost-effectiveness analysis, we calculated the effectiveness of the primary outcomes, the costs, and the incremental cost-effectiveness ratio (ICER) for each group. The total cost calculations were made from a societal perspective as recommended for the evaluation of health care programmes [33]. This means that costs were calculated independently of those who bear these costs and receive benefits.

To calculate the total costs, we measured the costs of consuming health care (six items) and the costs of production loss (seven items) by an adapted version of the Trimbos/iMTA questionnaire for costs associated with psychiatric illness [52]. Furthermore, we measured the travelling distance to visit the OP, to calculate out-of-pocket costs. Except for the duration of sick leave, the items originate from the TiC-P questionnaire, which in turn originates from the Short-Form Health and Labor Questionnaire (SF-HLQ). We calculated the costs of production loss for each group by multiplying respectively the mean duration of sick leave and the mean production loss without sick leave (three items) with the mean net income from paid work (one item). The reported net income will be validated with national data of income related to age and gender. If these data can not be considered valid or the income data of too many patients are unknown, we will use national data of average production value per worker related to age and gender.

To calculate the costs of production loss due to sick leave, we used the friction-costs method [33]. This method calculates the loss of production until the vacancy because of sick leave is resolved.

To calculate the costs of production loss without sick leave, we averaged the costs calculated by the method of Osterhaus and the costs calculated by the HLQ method, as there is a lack of a golden standard. These two methods give an overestimation and underestimation of the real costs, respectively. In the Osterhaus method, production loss is based on the number of days working with hindrance because of health complaints and an estimation of the efficiency during those days. In the HLQ method, production loss is based on the number of hours one needs to catch up with the work one was not able to do because of health complaints.

The costs of health care were calculated by multiplying the number of contacts and visits to health care professionals, the number of days receiving ambulant or institutionalized treatment, and the use of medication, with the prices of health care according to the guidelines for prices in the Netherlands [53].

We did not correct for inflation and did not discount costs as the follow-up period of the study is only one year [33]. All the costs are expressed in euros for the relevant reference year 2006, 2007, or 2008, the year in which the costs were generated.

### Independent measures

We measured job and personal characteristics and working conditions at baseline as independent measures. As job characteristics, we assessed profession, industry, number of contract hours, type of contract, and years employed with the employer. As personal characteristics, we measured age, gender, civil status, and educational level. As working conditions, we assessed work pace and workload, emotional strain, decision latitude, autonomy, social support colleagues and social support supervisor, ruminating, enjoyment of the work, and job insecurity. Furthermore, we measured the expected duration of sick leave, diagnosis, psychological complaints, coping style, self-efficacy of return to work, and work load at baseline, as independent measures. We considered all of these parameters to be potential prognostic factors for the duration of sick leave.

### Statistical analysis

#### Effect evaluation

The time until full return to work is a primary and the time until partial return to work a secondary outcome. These outcomes are based on time-to-event data and therefore a survival analysis is appropriate. An intention-to-treat analysis will be performed by means of multilevel analysis, with OPs at the primary hierarchical level and patients at the secondary hierarchical level. If suitable software for multilevel analysis is not available, we will use a regular Cox's multivariate regression analysis to answer the first and second research questions. We will use the 2 ll likelihood ratio statistical test for testing differences in the outcome scores between groups. The duration of sick leave will be analysed by multilevel ANOVA. To test differences in the prevalence rates, we will use the Mann Whitney U test.

Except for the time until partial return to work secondary outcome scores will be analysed by multilevel MANOVA.

#### Cost-effectiveness evaluation

To test statistically the differences in cost-effectiveness between groups, we will use the Net Monetary Benefit (NMB) as a parameter instead of the ICER, as this gives us the opportunity to test differences in cost-effectiveness with a regular statistical test. Therefore, we will re-scale the difference in effect between the two programmes into monetary value by using the threshold willingness-to-pay for a unit of effect, and the difference in costs between the two programmes is subtracted from this value. To test the differences, we will use the non-parametric bias-corrected accelerated bootstrapping test. We will also present a cost-effectiveness plane and an acceptability curve, which show the probability that the RTW-E programme is more cost-effective than the CAU. In order to determine the relative impact of changes in values of different parameters on the cost-effectiveness calculation, we will perform one-way and multi-way sensitivity analyses.

## Discussion

The results of this study will provide input for evidence-based treatment options for workers on sick leave due to common mental complaints. We expected the return-to-work programme based on the principles of exposure in vivo (the RTW-E programme) for workers on sick leave due to common mental complaints to be more effective in enhancing full return to work compared with care as usual (CAU). CAU in this study is a rather stringent control condition, as Van der Klink et al. [15] found a reduced duration of sick leave (21 days) of this intervention related to the CAU at that time. Nevertheless, we assumed the RTW-E programme to be robust enough to generate significant and clinical relevant differences in the duration of sick leave between groups. Employing a CAU control does have the benefit of practical relevance. However, such a control group could have reduced the contrast between both groups and may have reduced the potential difference in effect size between groups as a consequence.

### Strengths and weaknesses

#### Strengths

In this field study, we regularly measured outcome – every three months during a year – which enabled the detection of relevant changes in clinical status. We used a diagnostic interview to include or exclude patients, aiming to standardize the diagnostic process. To calculate the cost-effectiveness, we incorporated, besides losses due to sick leave and the costs of using health care facilities, also productivity losses during work (presenteeism). These aspects of the study increase internal validity.

The external validity of the results of the cost-effectiveness analysis is improved by the choice of a societal perspective of the analysis as we can disaggregate the calculated cost-effectiveness from the societal perspective to other levels, such as cost-effectiveness for employers [33]. Moreover, external validity is high as this study can be considered a pragmatic randomized trial, which means the RTW-E programme is evaluated in the context and reality of circumstances in which OPs operate daily.

To stimulate compliance with the RTW-E programme, we offered OPs in the intervention group a two-day training before inclusion started and three follow-up sessions during the inclusion period. The follow-up sessions offered OPs the opportunity to discuss difficulties and possible solutions in applying the interventions of the RTW-E programme with colleagues. A higher compliance with the RTW-E programme increases the level to which differences between groups could be attributed to the RTW-E programme.

#### Weaknesses

In this study, we selected patients on sick leave via OPs, and OPs were randomized instead of patients. Although this is an efficient way to include patients in the occupational health field, we may have introduced selection bias by doing this. Furthermore, OPs were not blinded to the RTW-E programme as they were trained in the new programme. This could also have introduced selection bias. These aspects of selection bias could possibly exaggerate the effect size of the study and thereby decrease internal validity. The OPs could have had a tendency to invite favourable patients if they reasoned that the RTW-E programme was appropriate for them, instead of inviting and including every patient with a CMD [54].

Although we monitored compliance with the RTW-E programme and judged the quality of the filled-in homework assignment forms, the counselling sessions remained mainly a black box, as we did not audio- or videotape treatment sessions and based our judgement of compliance on a list of treatment criteria.

## Competing interests

The authors declare that they have no competing interests.

## Authors' contributions

JvdK conceived the rationale of the intervention. EN and KN developed the intervention. KN, JvdK and FvD participated in the design and co-ordination of the trial. EN carried out data collection and wrote the manuscript. All authors provided comments on the drafts of the manuscript and have read and approved the final version.

## Pre-publication history

The pre-publication history for this paper can be accessed here:


